# The application of photoelectrocatalysis in the degradation of rhodamine B in aqueous solutions: a review

**DOI:** 10.1039/d2ra04236c

**Published:** 2022-09-21

**Authors:** Tunde Lewis Yusuf, Benjamin O. Orimolade, Daniel Masekela, Bhekie Mamba, Nonhlangabezo Mabuba

**Affiliations:** Department of Chemical Sciences, University of Johannesburg Doornfontein, P.O. BOX 17011 2028 Johannesburg South Africa nmabuba@uj.ac.za; Institute for Nanotechnology and Water Sustainability (iNanoWS), College of Science, Engineering and Technology, University of South Africa Private Bag X6, Florida Science Campus 1709 Johannesburg South Africa

## Abstract

The pollution of the water environment by industrial effluents is an ongoing challenge due to the rate of industrialisation and globalisation. Photoelectrocatalysis (PEC), an electrochemical advanced oxidation process, has proven to be an effective method for removing organics from wastewater. Photoelectrocatalysis is environmentally benign, cost-effective and easy to operate. In this present review, we examine the recent progress in the removal of rhodamine B dye, a common constituent of textile effluent released into the environment, through photoelectrocatalytic degradation. We present a detailed discussion on the use of different kinds of unmodified and modified photoanodes that have been explored for the photoelectrocatalytic removal of this dye. More importantly, discussions are presented on the mechanisms and kinetics of the degradation of rhodamine B dye using these photoanodes. Hence, this review will be beneficial for researchers in developing future projects in the area of wastewater treatments through photoelectrocatalysis.

## Abstract 2

The pollution of the water environment by industrial effluents is an ongoing challenge due to the rate of industrialisation and globalisation.

## Graphical abstract

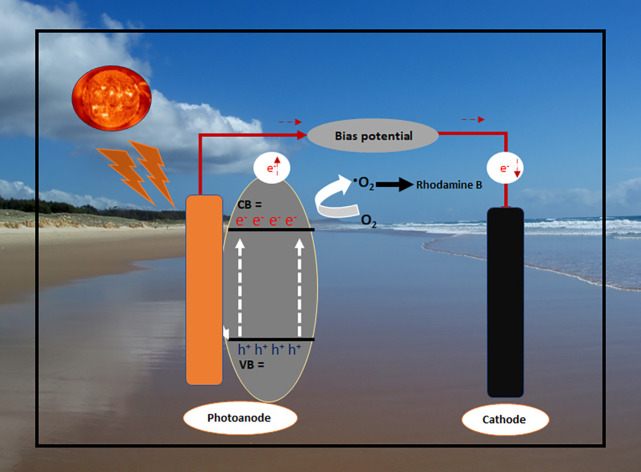

## 1 Introduction

There are many challenges presently limiting the quality of life and the environment. Among these challenges, the issue of water pollution has remained a significant concern as it is mainly responsible for water scarcity and water stress in both developed and developing societies. The problem of water pollution in recent years can be directly linked to an exponential increase in the rate of urbanization and industrialization.^1^ Water pollution can be from natural sources, which could result from natural phenomena such as acid rain or natural disasters like volcanic eruptions. Still, the bulk of water pollution originates from anthropogenic sources such as improper disposal of industrial effluents and household wastes which ultimately end up in surface water and groundwater.^2^ It has been noted that water pollutants are diverse, and they include a wide range of heavy metals such as iron, lead, arsenic and selenium.^3^ However, many pollutants detected in water are organic in nature and belong to numerous classes. Examples of such organic pollutants include pesticides originating from agricultural activities, pharmaceuticals and personal care products emanating from pharmaceutical industries, hospitals and households, and organic dyes originating from textile industries.^4^

The textile sector remains a robust sector responsible for more than 800 tons of synthetic dyes yearly.^5^ Unfortunately, more than 10% of this dyestuff ends up in the environment as constituents of untreated or improperly treated effluents.^6^ This is because when dyes are applied to fabric materials, not all the dye molecules are retained by the materials, and specific loss of dyes has been estimated to range between 2% and 50% for basic dye and reactive dyes, respectively.^7^ In textile industries, the effluents could be in liquid or solid forms consisting of chelating agents, emulsifying oils, softening agents, surfactants, acids, promoting agents and dyes used in the dyeing process. Hence, the effluent is characterized by high pH, intense colour, suspended solids and dissolved solids.^8^ Unfortunately, textile dyes in the effluent have been associated with many environmental and living organism problems. For instance, they prevent the growth of aquatic organisms and limit the self-purification of water.^9,10^ Additionally, when humans and animals are exposed to water polluted with textile dyes, they face many health hazards. Thus, the negative impacts of water pollution by textile dyes are related to health, economic and environmental issues. Therefore, efficient and sustainable solutions must be provided to eliminate these textile dyes from polluted water.

Since textile dyes have been established to persist as non-biodegradable organics in the environment and traditional wastewater treatment options have proven ineffective, concerted efforts have been dedicated to developing novel methods capable of completely eliminating textile dyes from wastewater. Earlier researchers embraced the adsorption process as a simple and cost-effective method for the removal of dyes from wastewater, and they explored the use of several materials as suitable adsorbents. Examples of such adsorbents include metal oxide nanoparticles,^11,12^ carbonaceous materials^13^ and low-cost biosorbents derived from agricultural wastes.^14,15^ Unfortunately, the adsorption process has reported limited success in this regard, and it also suffers the problem of the generation of secondary pollutants.^16^ Recently, methods based on advanced oxidation processes (AOPs), which largely depend on the use of reactive oxygen species (ROS) such as hydroxyl radicals and superoxide radicals as powerful oxidants, have been identified to be capable of total removal of textile dyes from wastewater.^17^ A prominent example of AOPs is the photocatalysis process which involves the use of metal oxide semiconductors such as TiO_2_,^18^ ZnO,^19^ BiVO_4_,^20^ Bi_2_WO_6_,^21^ Cu_2_O^22^ and WO_3_.^23^ However, the problems of rapid recombination of charge carriers limit the success of photocatalytic removal of textile dyes in wastewater. This led to the development the of photoelectrocatalytic degradation process, whereby the application of bias potential significantly reduces the issue of spontaneous recombination of photogenerated charge carriers in the semiconductors.^24^

Tremendous success has been recorded in applying the photoelectrocatalysis (PEC) process for removing textile dyes from wastewater using various kinds of photoanodes. Hence, this review aims to present a critical discussion on the recent advancement in the use of a wide range of anodic materials to remove a commonly used textile dye, rhodamine B, in wastewater. It is worth noting that the removal of rhodamine B has been the subject of many research studies due to its unpleasant effects when found in water environments. In fact, the amount of literature available on the removal of rhodamine B has prompted many researchers to write review articles to understand the progress and intricacies of eliminating rhodamine B from wastewater. For example, Al-Gheethi *et al.* published a review on the removal of rhodamine B using adsorbents made from agricultural wastes.^25^ In another recently published work by Al-Buriahi *et al.*, a critical discussion on the use of nanoparticle photocatalysts for the removal of rhodamine B from textile wastewater was presented.^26^ However, the focus of this review is on the use of PEC degradation technique for the removal of rhodamine B from wastewater. A robust discussion is presented on different novel materials that have been employed as photoanodes as well as their preparation. The factors affecting the PEC degradation processes were succinctly enumerated, along with the kinetics and mechanisms of the process. Hence, this review article will benefit scientists concerned with finding sustainable ways to remove organic dyes from polluted water.

## 2 Rhodamine B dye

**Fig. 1:**
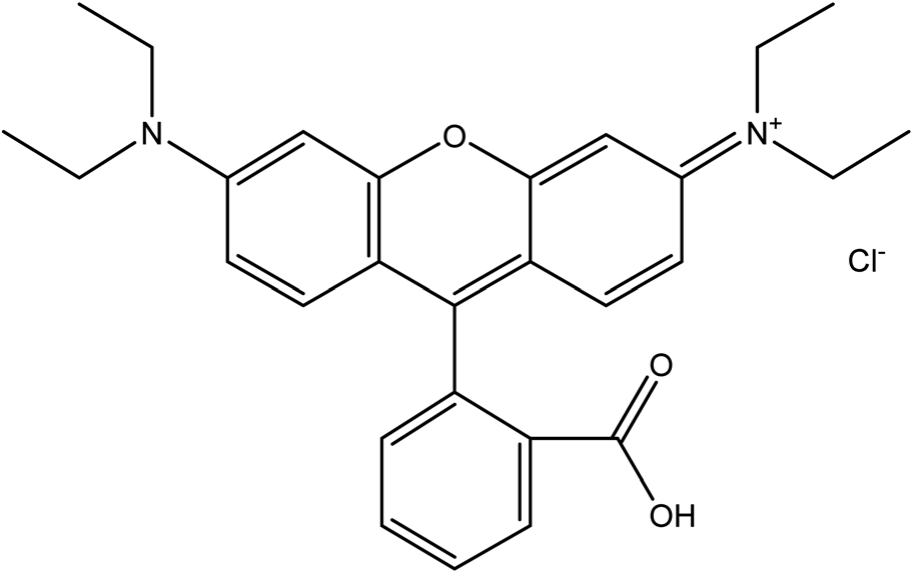
Chemical structure of rhodamine B.

Rhodamine B dye is a common water-soluble dye that is widely used in textile industries for dyeing wool fabrics. Additionally, in other industries such as paper, plastic, printing, biomedical and leather industries, rhodamine B is commonly employed as a colouring agent, photosensitizers, water tracer, fluorescent markers for microscopic structural analysis and biological stain in biomedical research.^27–29^ Hence, it is a common constituent of effluents emanating from these industries. Rhodamine B, one of the oldest synthetic dyes employed in food and fabric dyeing industries, is an amphoteric dye belonging to the broad class of xanthene dyes. It is one of the most environmentally benign xanthene basic dyes.^25^ According to IUPAC nomenclature, rhodamine B is called *N*-[9-(*ortho*-carboxyphenyl)-6-(diethylamino)-3*H*-xanthen-3-yli-dene] diethyl ammonium chloride. It has a molecular weight of 479.02 g mol^−1^ and exhibits maximum absorbance at a wavelength of 554 nm.^30^ It is a highly water-soluble basic red dye, and its dissolved form can readily be extracted using ethanol or butanol. Generally, the preparation of rhodamine B depends on the modification of amino groups of xanthene compounds *via* a route that connects the units to form a glycosidic bond.^31^ The typical chemical structure of rhodamine B (C_28_H_31_ClN_2_O_3_) is shown in Fig. 1. In a neutral condition, the closed/nonfluorescent spirolactam form and the open/fluorescent form of rhodamine B exist in equilibrium. However, under basic conditions, the closed form, which is colourless, is favoured while the open form is favoured under acidic conditions.^32^

Due to its vast applications in many industries that discharge partially treated effluents into the environment, it is not surprising to find rhodamine B in the water environment. Unfortunately, the water pollution by rhodamine B has been linked to many environmental issues and health risks in humans and animals. Even when the concentration of rhodamine B in water is very low (approximately 1.0 mg L^−1^), it impacts a strong colour on the water, making it unsuitable for domestic use and consumption.^33,34^ Aquatic organisms suffer greatly from the presence of rhodamine B in surface water as it significantly prevents the penetration of light to algae and other plants in the water, hindering the photosynthesis process. In a study conducted by Kooh *et al.*, it was observed that rhodamine B is hazardous to *Cyprinodon variegatus* at the lethal concentration of 84 mg L^−1^.^35^ Additionally, in water contaminated by rhodamine B, it has been noted that the population of phytoplankton and zooplankton is very low.^36^

Furthermore, contact with rhodamine B can damage the eyes and cause irritation and severe oral noxiousness in humans.^33^ However, humans are susceptible to greater risks when they ingest rhodamine B. Studies in the field of medicine have confirmed that rhodamine B is mutagenic and carcinogenic in nature, causing developmental and simulation toxicity in animals and humans.^37^ It has also been associated with the disruption of central nervous systems and other vital organs, including the liver, brain and kidney.^38^ Hence, its use in food processing industries has been prohibited.^39^ Nevertheless, it is paramount that effluents originating from industries dealing with rhodamine B are subjected to suitable wastewater treatment methods to absolutely remove rhodamine B from them before being discharged into the environment. This is very important since rhodamine B has reasonably good phostability^40^ and hence cannot be easily degradable in the environment with exposure to direct sunlight.

In the quest to tackle the problem of water pollution due to the presence of rhodamine B dye, several wastewater treatment options have been explored for decolourisation and degradation of rhodamine B in water. These treatment options include processes based on physical, biological or chemical principles. A prominent example of these treatment options is the adsorption process which is very economical and environmentally friendly. This adsorption of rhodamine B has been ongoing for decades. In as much as it has its demerits, the research studies in this are still on the increase, and the choice of adsorbents is evolving. Recently, attention has been given to improving adsorption operation by incorporating membrane separation technology. For example, Wang *et al.* studied the removal of rhodamine B through an adsorptive membrane using a composite material consisting of graphene oxide–polydopamine coupled with polyethersulfone–sulfonated polyethersulfone membrane.^41^ The adsorption process was found to be chemisorption, and an impressive adsorption capacity of 26.34 mg g^−1^ was reported for the composite material. Similarly, in another study by Gharbani and Mehrizad, rhodamine B was removed through an adsorptive membrane process.^42^ The material employed was a composite of graphitic carbon nitrides, chitosan and polyvinylidene fluoride. The maximum adsorption capacity for rhodamine B was reported to be 33.46 mg g^−1^ which also corresponds to 72.74% removal of the dye. However, due to inherent limitations of the adsorption process, other wastewater treatment options particularly advanced oxidation processes.

Photocatalysis, an example of AOPs, has been extensively studied to remove rhodamine B in an aqueous solution. The process is also easy to operate and environmentally friendly. However, unlike the adsorption process, photocatalysis can completely mineralise rhodamine B molecules in aqueous solutions. Though numerous kinds of pristine metal oxides semiconductors have been employed for the photocatalytic removal of rhodamine B, the majority of the recent photocatalysts are composites of two or more metal oxides with heterojunctions. For example, Harish *et al.* reported the photocatalytic removal of rhodamine B dye using a binary composite of molybdenum disulfide and nickel disulfide (MoS_2_/NiS_2_).^43^ The nanocomposite consisting of several spherical particles with nanosheets was prepared through the hydrothermal method at a temperature of 180 °C for 24 h. When the composite material was applied for photocatalytic removal of rhodamine B under visible light illumination, the percentage degradation was found to be approximately 91%. In another study reported by Truong *et al.*, nanocomposite of ZnO and CuO was successfully applied for the photocatalytic degradation of rhodamine B.^44^ The material was prepared using the sol–gel method followed by calcination. The material achieved 98% removal of rhodamine B through photocatalysis, and this was higher than the percentage recorded using pristine ZnO and CuO. Other composites that have been recently reported for the photocatalytic degradation of rhodamine B include ZnO/ZnFe_2_O_4_,^45^ BiOI/MgCrO_4_,^46^ ZnO/PbCrO_4_ (ref. 47) and C_3_N_4_/CoWO_4_.^48^

Another wastewater treatment option that has been employed for the removal of rhodamine B in water is the photo-Fenton process. This process is similar to classic Fenton, where H_2_O_2_ and ferrous ions are used to produce hydroxyl radicals to oxidise organics.^49^ However, unlike the typical Fenton process where ferrous salt is needed, in the photo-Fenton process, the semiconductor photocatalyst also serves as the source of the ferrous ion and a light source is also used. This strategy helps minimize the rapid recombination of photogenerated electron–hole pairs in the photocatalyst. In a study by Welter *et al.*, degradation of rhodamine B was achieved through a photo-Fenton process using a composite of chitin biochar and ZnFe_2_O_4_.^50^ This material was prepared using the sol–gel method, and the presence of biochar in the composite promoted efficient charge separation in the ZnFe_2_O_4_. Impressively, after 1 h, 100% discolouration of rhodamine B was achieved in the photo-Fenton process. In another study by Zhang *et al.*, iron–copper-supported montmorillonite was used for the photo-Fenton removal of rhodamine B dye. A two-step strategy of impregnation and calcination was adopted for the preparation of the catalyst. After 90 min, 98% discolouration of rhodamine B was recorded using the material. Due to the great prospect of Fenton processes for degradation of organics, the electro-Fenton process has also been studied to remove rhodamine B dye.^51,52^

## 3 Photoelectrocatalytic degradation: fundamentals

Photoelectrocatalytic oxidation is an example of an advanced electrochemical oxidation process with applications in sensing, water splitting and degradation of organics. The foundation of PEC process can be traced to the pioneering work on photoelectrochemistry by Brattain and Garrett.^53^ Later, the concept of water splitting in photoelectrochemistry was discovered by Fujishima and Honda in 1972 where they employed TiO_2_ as the photoanode.^54^ Generally, PEC process combines the photocatalytic process synergistically with electrochemical oxidation. Typically, in PEC degradation process, a photoactive material, usually a metal oxide semiconductor, is employed as the anode (photoanode) and then irradiated with light of suitable wavelength along with the passage of electrical energy through the electrode.^55^ When exposed to light, the photoanode absorbs photons and excites electrons from the semiconductor's valence band to its conduction band, much like in traditional heterogeneous photocatalysis. As a result, holes are formed, which are oxidants capable of degrading organic molecules in the solution. Additionally, the holes produce hydroxyl radicals from their interactions with water molecules at the anode surface. The hydroxyl radicals which are strong oxidants further oxidize the organic molecules to carbon dioxide, water molecules or lower chain hydrocarbons. Since this process in PEC system is similar to the mechanism of photocatalysis, it is expected that the issue of spontaneous recombination of photogenerated electron–hole pairs will limit the efficiency of the oxidation process. However, the application of external potential provides a force to drive away electrons from the photoanode resulting in the promotion of charge separation and reduction the in combination of photogenerated charge carriers.^56^ It is also important to note that the electrons facilitate the formation of other oxidants within the PEC system. Specifically, apart from holes and hydroxyl radicals, other oxidizing species in PEC system include superoxide and hydroperoxyl radicals. Eqn (1)–(5) summarize the formation of these oxidants from the photoanode.^55,57^

1 *hv* + semiconductor → h^+^ + e^−^

2 h^+^ + H_2_O → ˙OH + H^+^

3 e^−^ + O_2_ → ˙O_2_^−^

4 ˙O_2_^−^ + H^+^ → ˙HO_2_

5 2˙HO_2_ → H_2_O_2_ + O_2_

Even though PEC degradation incorporates elements of photocatalysis and electrochemical oxidation, it has some outstanding advantages over the two processes. Though, unlike some AOPs, the PEC degradation process requires the use of electrical power sources like potentiostat which may be expensive and require special expertise, the desirable advantages of PEC process over other AOPs make the process attractive. In addition to efficient charge separation that is obtainable in PEC, it offers better and easy reusability of photoactive material than in photocatalysis. In photocatalysis, the powdered or granular photocatalysts often require regeneration with chemical reagents for reuse. However, in PEC degradation system, the photoactive materials constitute compact electrodes and hence can simply be rinsed with deionised water, dried and reused. This is more economical and time-efficient. Furthermore, when PEC system is compared with the electrochemical oxidation process (anodic oxidation), a prominent advantage is that lower bias potential is sufficient for the mineralisation of organics in PEC system, unlike anodic oxidation, which requires a higher magnitude of applied cell potential.^58^

In PEC degradation, the choice of the photoanode material plays a crucial role in achieving better efficiency. Like in photocatalysis, the semiconducting metal oxides that have been mostly explored as suitable photoactive materials for the anode in PEC degradation systems are TiO_2_ and ZnO.^59–62^ These systems have been extensively adopted for the mineralisation of a wide range of organics, including pesticides, dyes, pharmaceuticals and phenolic compounds.^63,64^ The major hindrance to using TiO_2_ and ZnO as photoanodes is their relatively large band gap energies (∼3.5 eV for TiO_2_) which dictate the use of UV irradiation for better excitation electrons.^56^ However, humans are at risk when exposed to UV irradiation.

Additionally, the high cost associated with the use of TiO_2_ and ZnO photoanodes due to the cost of UV light sources is a key demerit. Hence, researchers have explored numerous strategies to make TiO_2_ and ZnO absorb photons within the visible light region as this will encourage the use of sunlight as the source of irradiation. Such strategies include tuning morphologies and doping with both non-metal and metal elements. For example, boron, nitrogen, graphene, nitrogen and fluorine are common non-metal dopants that have been used to promote visible light absorption in TiO_2_.^65–67^

In the last few years, the use of visible light-active metal oxide semiconductors as photoanode materials in PEC has been embraced. These materials have narrow band gaps and can easily absorb photons for excitation within the visible light spectrum, and their use encourages the adoption of direct sunlight as a suitable light source for PEC degradation systems. Examples of visible light active that are common as photoanodes for PEC degradation of organics include g-C_3_N_4_,^68^ BiVO_4_,^69^ WO_3_,^70^ MoS_2_,^71^ Bi_2_WO_6_,^72^ and Ag_3_PO_4_.^73^ These materials have also been used to improve the performances of TiO_2_ and ZnO.^74–76^ As a result of the narrow band gap energies of these visible-light active semiconductors, they are more susceptible to the spontaneous recombination of photogenerated electron–hole pairs, which could significantly decrease the efficiencies of their PEC systems and, therefore, their use in pristine form is not encouraged. In order to combat this challenge, doping strategy has been used.^77,78^ However, the construction of semiconductor–semiconductor heterojunction has recorded more success in this regard. A heterojunction is formed when two semiconductors of unequal band gap combine in such a way that results in band alignment, which enables the separation of photogenerated holes to the valence band and electrons to the conduction band.^79^ Numerous examples of heterostructured photoanodes consisting of at least one visible light-responsive semiconductor have been reported for the PEC degradation of organics in wastewater. Examples include BiVO_4_/WO_3_,^80^ Fe_2_O_3_/Bi_2_WO_6_,^81^ Cu_2_O/Fe_2_O_3_ (ref. 82) and BiVO_4_/Ag_2_S.^83^

## 4 PEC degradation of rhodamine B

The successful degradation of rhodamine B dye has been achieved through PEC oxidation using different photoanodes. Some of these photoanodes consisted of pristine metal oxide semiconductors, while others were doped of composite with heterojunctions. For the composite materials, the PEC efficiency is often compared to the corresponding pristine materials. The conducting substrates often employed are fluorine-doped tin oxide glass (FTO), titanium sheet or exfoliated graphite.

### 4.1 Unmodified photoanodes

**Fig. 2:**
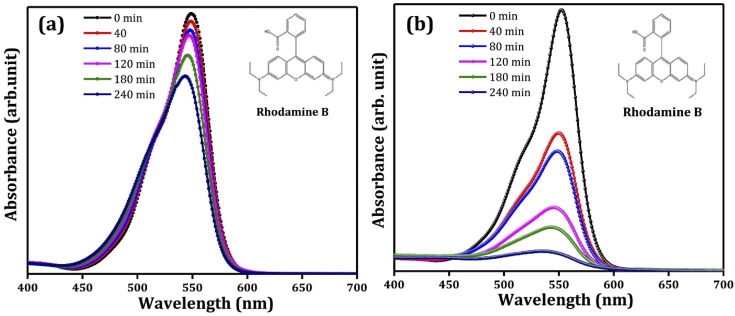
Typical UV/visible spectra of removal of rhodamine B through (a) photocatalysis and (b) PEC degradation (reproduced from ref. 84 with permission from [Elsevier B.V.], copyright [2022]).

Pristine metal oxide semiconductors have been supported/immobilized on different conducting substrates for PEC degradation of rhodamine B. In a study conducted by Pedanekar *et al.*, Bi_2_WO_6_ prepared on FTO glass was adopted as a suitable photoanode.^84^ The photoanode was prepared by deposition of thin films of Bi_2_WO_6_ on FTO glass through spray pyrolysis, and the volume of the spray solution was varied between 50 ml and 80 ml to obtain different Bi_2_WO_6_/FTO photoanodes. The prepared Bi_2_WO_6_ has an orthorhombic crystal structure and a porous morphology consisting of several interconnected microparticles. The Bi_2_WO_6_ electrode obtained with 70 ml spray solution showed better optical and electrochemical performances. The photoanode was then applied to degrade 30 mg L^−1^ rhodamine B dye solution under visible light irradiation with 0.7 V applied potential. After 2 h, 94% removal of rhodamine B was achieved. The degradation pattern as observed using UV-vis spectrophotometer are shown in Fig. 2(a and b). This percentage removal was over three times greater than the values obtained with photocatalysis and electrochemical oxidation using the same electrode. Additionally, the rate of degradation of the dye was fastest with PEC degradation. Similarly, in the works of Amaterz *et al.*, efficient degradation of rhodamine B was recorded using a photoanode made up of barium hydrogen phosphate films electrodeposited on FTO glass.^85^ The PEC degradation of 6.75 mol L^−1^ of rhodamine B using the electrode resulted in 99% removal within just 7 min. In order to establish that the electrode achieves mineralisation of the dye and not just discolouration, the total organic carbon (TOC) value of the treated dye solution was measured, and an impressive value of 83% was recorded. The researchers also noted that coupling photocatalysis and electrocatalysis in the PEC degradation led to a synergistic effect in dye removal. These results clearly show that photoanodes consisting of pristine photoactive materials on FTO glass are capable of degrading rhodamine B molecules in PEC systems.

**Fig. 3:**
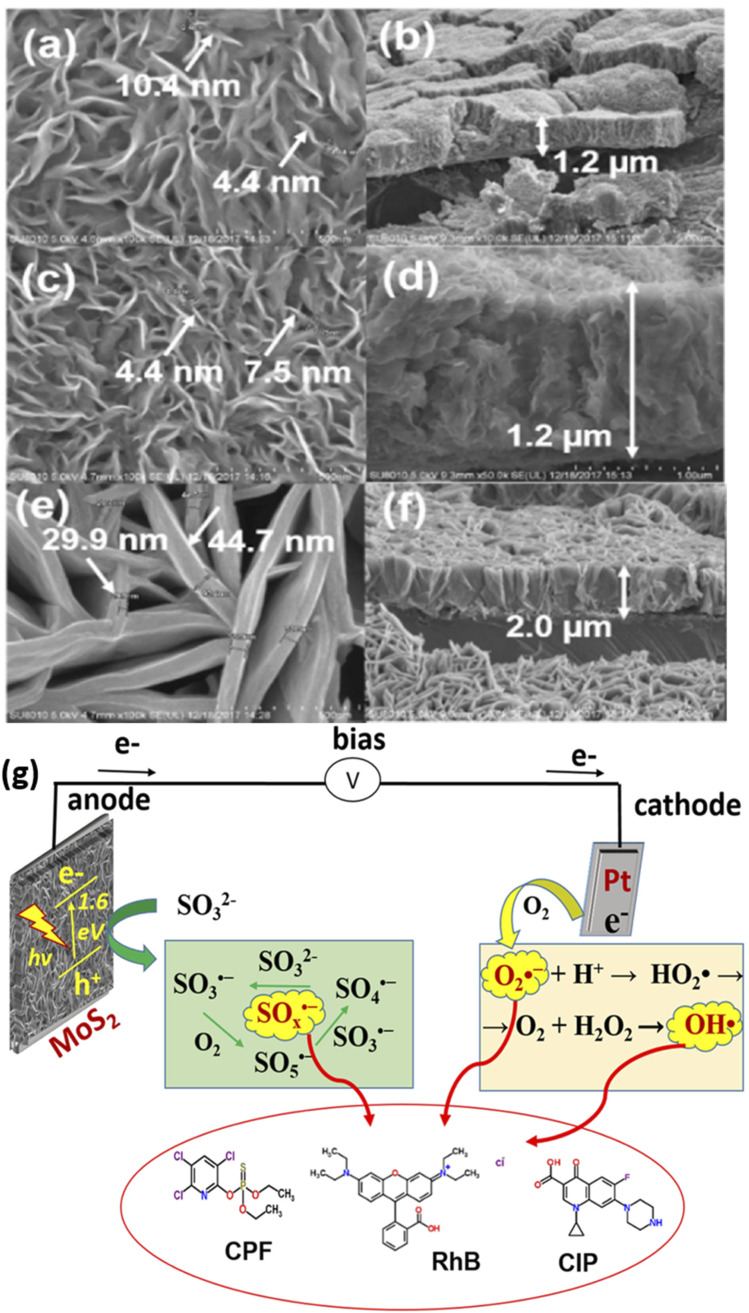
SEM images of as-prepared MoS_2_ nanoflakes (a and b), MoS_2_ (300 °C) nanoflakes (c and d) and MoS_2_ (800 °C) (e and f) vertically aligned on Ti foil ((a, c and e) from top view; (b, d and f) from side view) (reproduced from ref. 86 with permission from [Elsevier B.V.], copyright [2022]) and illustration of PEC *in situ* generation of oxysulfur, superoxide and hydroxyl radicals (g) (reproduced from ref. 87 with permission from [IWA Publishing], copyright [2021]).

Furthermore, photoactive materials have also been prepared on titanium sheet or foil for the PEC removal of rhodamine B dye. For example, Zhou *et al.* reported the PEC removal of rhodamine B dye using a photoanode consisting of MoS_2_ nanoflakes prepared on titanium foil.^86^ The electrode was formed by growing MoS_2_ films on titanium foil in a Teflon-lined reactor through hydrothermal synthesis at 180 °C for 24 h. Additionally, the MoS_2_ nanoflakes were further annealed at temperatures of 300 °C and 800 °C. The results from the XRD analysis revealed that the crystallinity of the MoS_2_ increased with an increase in annealing temperature. The MoS_2_ nanoplatelets were observed to be vertically aligned to the titanium foil, as shown in Fig. 3(a–f). The MoS_2_ PEC system was applied to remove rhodamine B dye with an initial concentration of 1.0 mg L^−1^. After a 2 h reaction time with an applied potential of 0.5 V, the complete decolourisation of the rhodamine B dye solution was almost achieved. The primary oxidants in the MoS_2_ PEC system were found to be hydroxyl radicals as the degradation efficiency reduced significantly with the addition of *tert*-butyl alcohol. In a similar study conducted by the same group, it was observed that several reactive species such as superoxide radicals, oxysulfur radicals and hydroxyl radicals were produced in the MoS_2_ PEC system for degradation of rhodamine B.^87^ The mechanism of this degradation process showing the generation of these radicals is depicted in Fig. 3g. In another study by Shao *et al.*, Ru_*x*_Zn_1−*x*_O was prepared on titanium plates and employed as a photoanode for PEC degradation of 20 mg L^−1^ rhodamine B dye solution.^88^ Within 2 h, the percentage degradation of the dye was 97%, with an applied potential of 2.5 V. An attractive property of these electrodes prepared on titanium sheet or foil was that they offer excellent stability.

Though unmodified photoanodes have been identified as been capable of removal of rhodamine B through PEC oxidation, the efficiency of the process is significant hindered as a result of rapid recombination of photogenerated electron–hole pairs. This problem can be overcome through appropriate modification of the material which may involve morphology control, doping and formation of heterojunction. These approaches seek to promote efficient charge separation in the photoanode which can then translate to improved PEC degradation efficiency. Another approach to counter the problem is the choice of conducting substrate. For instance, exfoliated graphite can be employed because of its dual function of acting as electron sinking and being a good conductor. Titanium sheet can also be anodized to TiO_2_ nanotubes which can form heterojunction with other metal oxide semiconductors.

### 4.2 Doped photoanodes

In recent studies, modified photoanodes are often employed for PEC degradation of rhodamine B to overcome the problem of rapid recombination of photogenerated electron–hole pairs prevalent in pristine photoanodes or to increase the visible light responsiveness of the photoanodes. The doping of metal oxide semiconductors with metals and non-metals has proven to be an effective strategy to increase the PEC efficiency of photoanodes in the degradation process. When a dopant is introduced into the structure of semiconductors, it can shift the band edge towards the visible region, induce oxygen vacancy, induce carrier trap density and increase the mobility of the charge carriers. This results in improve conductivity and photosensitivity.^89^ Many researchers have investigated the use of doped photoanodes for the PEC degradation of rhodamine B dye. For example, Reis *et al.* reported the use of nitrogen-doped ZnO as an ideal photoanode for PEC removal of rhodamine B.^90^ The photoanode was prepared through electrodeposition of ZnO on FTO glass under continuous nitrogen gas bubbling at different rates. Though doping the ZnO with nitrogen did not cause a change in the band gap energy of the ZnO, it did increase both the optical and photoelectrochemical properties of the photoanode. As evidence, the photocurrent response of the nitrogen-doped ZnO was around 70 μA cm^−2^ which was higher than of the pristine ZnO (48 μA cm^−2^). This was because doping with nitrogen promoted better electron mobility and reduced the electrical resistance of the ZnO. When the electrodes were applied for the PEC degradation of 0.48 mg L^−1^ rhodamine B solution, the catalytic efficiencies were 26% and 43% for pristine ZnO and nitrogen-doped ZnO electrodes, respectively. These findings confirmed that doping with non-metals such as nitrogen is an effective strategy to improve PEC efficiencies of photoanodes.

**Fig. 4:**
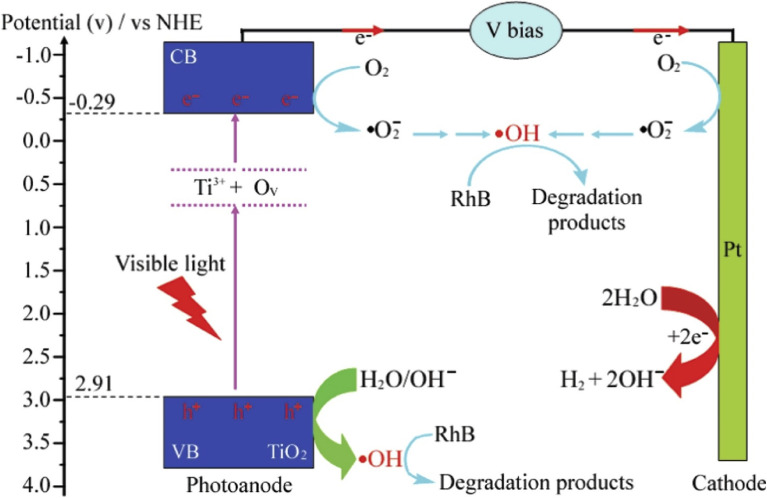
Schematic diagram of PEC degradation of rhodamine B (RhB) using Ti^3+^ self doped TiO_2_ (reproduced from ref. 93 with permission from [Elsevier B.V.], copyright [2016]).

In the case of TiO_2_, both metals and non-metals have been used as dopants to improve its PEC performance towards the mineralisation of rhodamine B dye. For example, Kiziltas reported the PEC degradation of rhodamine B using a photoanode consisting of TiO_2_ nanotubes codoped with boron and cobalt.^91^ The photoanode was prepared through anodization of titanium sheets in the presence of cobalt and boron salts, followed by calcination at 500 °C for 2 h. The dopants significantly decreased the band gap energy of TiO_2_ which resulted in better light absorption. Additionally, the photocurrent density recorded for the codoped TiO_2_ nanotubes was 2.1 mA cm^−2^ with applied potential of 0.6 V, which was almost seven times higher than the response of pristine TiO_2_ nanotubes (0.31 mA cm^−2^). The codoped photoanode achieved 95.5% PEC removal of 10 mg L^−1^ rhodamine B solution within 90 min while the undoped TiO_2_ nanotube degraded only 46.3% of the dye. This result evidently revealed that binary doping of photoanode can improve the PEC efficiency of the photoanode through enhanced electron mobility and better photon absorption. Selenium is another metal that has been employed as dopant for TiO_2_ nanotubes towards PEC degradation of rhodamine B.^92^ The selenium doped TiO_2_ achieved approximately 73% PEC removal of the dye while only 28% removal was recorded with the undoped material. The enhanced PEC performance was attributed improved visible light absorption due to the incorporation of selenium. Interestingly, self-doping approach with Ti^3+^ is another ideal way to extend the light responsiveness of TiO_2_. Wu *et al.* reported 10% and 99.9% PEC removal of rhodamine B within 50 min using undoped TiO_2_ and Ti^3+^ doped TiO_2_ photoanodes, respectively.^93^ The superior performance was linked to faster transport of photogenerated charge carriers, reduced recombination rate and improved light absorption due to Ti^3+^ self doping. As shown in Fig. 4, it can be seen that the Ti^3+^ introduced local states between the valence band and conduction band of TiO_2_ which enlarged the optical absorption of photoanode. Hence, doping photoanodes with metals is an effective method of improving PEC degradation of rhodamine B dye.

**Fig. 5:**
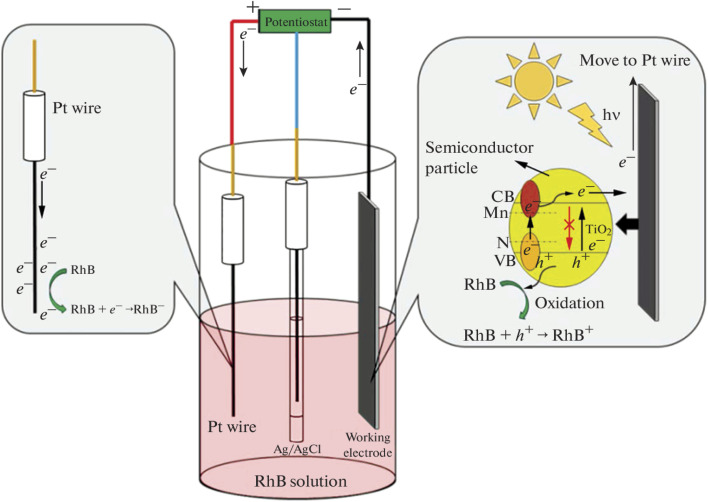
Presentation of PEC system for rhodamine B dye degradation at Mn, N doped TiO_2_ electrode (reproduced from ref. 94 with permission from [Allerton Press, Inc.], copyright [2022]).

Nurdin *et al.* also reported the PEC degradation of rhodamine B using a TiO_2_ electrode codoped with both metal and non-metal dopants.^94^ In their study, manganese and nitrogen were employed as suitable dopants to improve the performance of TiO_2_. A sol–gel method was employed to successfully doped the TiO_2_ films on a titanium sheet with the elements. The codoped TiO_2_ electrode showed better PEC efficiency under visible light illumination. Interestingly, the codoped electrode achieved 74.2% PEC removal of 0.5 mg L^−1^ rhodamine B dye under visible light illumination, whereas the best performance of the undoped TiO_2_ electrode under UV irradiation was 63%. Hence, doping with both manganese and nitrogen increased the visible light absorption of TiO_2_ by reducing the charge recombination and improving electron mobility. The mechanism of charge separation within the codoped electrode and the degradation of the dye molecules are depicted in Fig. 5. Such impressive PEC degradation of rhodamine B using TiO_2_ photoanode doped with both metal and non-metal dopants was also observed in the study reported by Kothavale *et al.*, where boron and nitrogen were used as the dopants.^95^

Considering the reports on the use of doped photoanodes for the removal of rhodamine B dye in aqueous solution through PEC oxidation, it can be inferred that numerous kinds of dopants can be adopted to improve the efficiency of the process. These dopants could be non-metals or metals which can easily extend the range of visible light absorbance of the photoanodes and also facilitate efficient charge separation within the photoanode. Hence, doped photoanodes offer better performance than undoped photoanodes. However, the choice of appropriate dopants and optimization of the doping process are very important to achieve a photoanode with good PEC efficiency.

### 4.3 Heterostructured photoanodes

Several types of photoanodes with semiconductor–semiconductor heterojunctions have been employed for the PEC degradation of rhodamine B dye in aqueous solutions. The use of heterostructured photoanode is attractive because they offer better efficiencies due to improve charge separations. The heterostructured photoanode may consist of p–n heterojunction or n–n heterojunction depending on the type of semiconductors used. For example, Jiang *et al.* reported the use of Bi_2_O_3_/WO_3_ photoanode with p–n heterojunction for the PEC degradation of rhodamine B dye.^96^ The composite films were prepared through hydrothermal synthesis onto FTO glass at a temperature of 160 °C for 6 h followed by calcination at 600 °C for 2 h. The optical characterisation results showed that heterojunction formation led to an increase in the absorption of visible light by the Bi_2_O_3_/WO_3_. Additionally, the composites photoluminescence intensity was weaker than the pristine Bi_2_O_3_ and WO_3_, which suggested a decrease in the recombination of charge carriers. The measurement of photocurrent responses further confirmed the improved charge separation in the Bi_2_O_3_/WO_3_ interface due to the formation of p–n heterojunction. Consequently, when the photoanodes were applied for the PEC degradation of 10 mg L^−1^, the highest removal efficiency (73.4%) was recorded with the Bi_2_O_3_/WO_3_ photoanode, which Bi_2_O_3_ and WO_3_ gave 33.3% and 25.9% respectively after 3 h. Hence the construction of p–n heterojunction with effective charge separation could effectively increase the PEC efficiency of removal of rhodamine B dye.

**Fig. 6:**
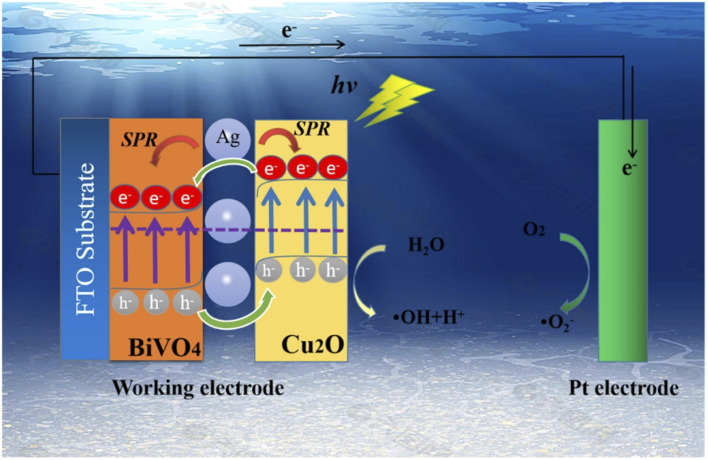
Schematic diagram illustrating the enhanced charge separation in Ag/BiVO_4_/Cu_2_O photoanodes in the PEC degradation of rhodamine B (reproduced from ref. 97 with permission from [Elsevier B.V.], copyright [2022]).

In another study by Ma *et al.*, a composite photoanode of BiVO_4_/Cu_2_O with p–n heterojunction decorated with silver nanoparticles was employed for the PEC degradation of rhodamine B.^97^ The BiVO_4_ was firstly prepared on FTO glass through hydrothermal synthesis, and silver nanoparticles were then deposited on it by a facile chemical water bath method. To obtain the p–n heterojunction, Cu_2_O were electrodeposited onto the prepared BiVO_4_/Ag/FTO at a potential of −0.5 V for 3 min. The morphology of the composite revealed that the photoanode consisted of compact BiVO_4_ nanosheets and Cu_2_O cubic nanocubes, which were agglomerated with Ag nanoparticles. The prepared BiVO_4_/Cu_2_O showed an improved response to visible light because of better charge separation through the formation of p–n heterojunction as shown in Fig. 6. Specifically, the absorption band edge of BiVO_4_ shifted from 512 nm to 549 nm in the BiVO_4_/Cu_2_O. The addition of Ag nanoparticles increased it to 568 nm due to the plasmon resonance effect. The percentage PEC removals of 5 mg L^−1^ rhodamine B with an applied potential of 1.2 V under visible light after 2 h were 30%, 69% and 86% using BiVO_4_, BiVO_4_/Cu_2_O and Ag/BiVO_4_/Cu_2_O photoanodes, respectively. The better removal achieved in the composite electrode was attributed to the resultant effect of enhanced charge separation and plasmon resonance effect. This further proved the suitability of p–n heterostructured photoanodes for removal of rhodamine B.

**Fig. 7:**
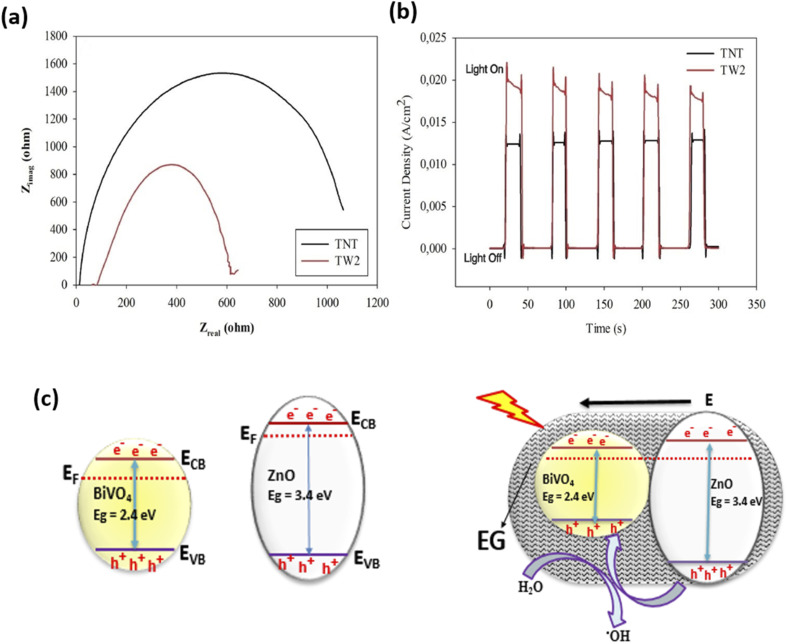
Nyquist plots for pristine TiO_2_ nanotubes (TNT) and WO_3_ deposited TiO_2_ electrode (TW2) (a); Transient photocurrent response of bare TiO_2_ and WO_3_ deposited TiO_2_ electrode (b) (reproduced from ref. 98 with permission from [Elsevier B.V.], copyright [2021]) and Mechanism of charge separation in BiVO_4_/ZnO n–n heterojunction (c) (reproduced from ref. 76 with permission from [Royal Society of Chemistry], copyright [2019]).

Improved PEC degradation of rhodamine B dye has also been achieved using photoanodes consisting of n–n heterojunction. Davaslıoğlu *et al.* report that a photoanode made up of WO_3_ and TiO_2_ nanotubes with n–n heterojunction were used for the PEC degradation of rhodamine B.^98^ The composite electrode was fabricated by electrodeposition of WO_3_ onto TiO_2_ nanotube arrays. As shown in Fig. 7(a and b), formation of heterojunction resulted in lower electric resistance and higher photocurrent responses. This was because the recombination of the photogenerated electron–hole was significantly reduced as the charge carriers were separated through the formation of heterojunction. Consequently, the heterostructured photoanode achieved about 70% PEC removal of rhodamine B within 30 min. This value was significantly higher than the performance of pristine TiO_2_ nanotubes suggesting that the formation of heterojunction is an effective way of increasing the PEC efficiencies of photoanodes. In another work by Orimolade *et al.*, a composite electrode consisting of BiVO_4_/ZnO with n–n heterojunction achieved 91% removal of rhodamine B through PEC process.^99^ The mechanism of the degradation process, as shown in Fig. 7c, revealed that band alignment between BiVO_4_ and ZnO resulted in the separation of photogenerated holes from the valence band of ZnO into that of BiVO_4_, which facilitated enhanced production of hydroxyl radicals resulting in better mineralisation of rhodamine B dye. Therefore, photoanodes with n–n heterojunctions are also ideal for the degradation of rhodamine B dye in an aqueous solution. It is also worth noting that the use of exfoliated graphite contributed to the efficiency of the system by serving as electron sink which enhanced photogenerated charge separation.

Evidently, heterostructured photoanodes have demonstrated better efficiencies in the PEC degradation of rhodamine B as compared to the use of pristine photoanodes. It is not surprising that many recent works are very particular about the fabrication of novel heterostructured photoanodes for PEC degradation of rhodamine B dyes and other organic pollutants. Though, doped photoanode have also recorded higher performance than undoped photoanode, the use of heterostructured is much attractive because of the possibility of coupling the strengths of two or more metal oxides semiconductors. For instance, a visible light active semiconductor can be used to improve the visible light responsiveness of UV active semiconductors. Additionally, improved charge separation is often accomplished with the formation of heterojunctions. It is therefore expected that more novel photoanodes for PEC degradation of rhodamine would still be investigated in the near future.

### 4.4 Stability of photoanodes in PEC degradation of rhodamine B

**Fig. 8:**
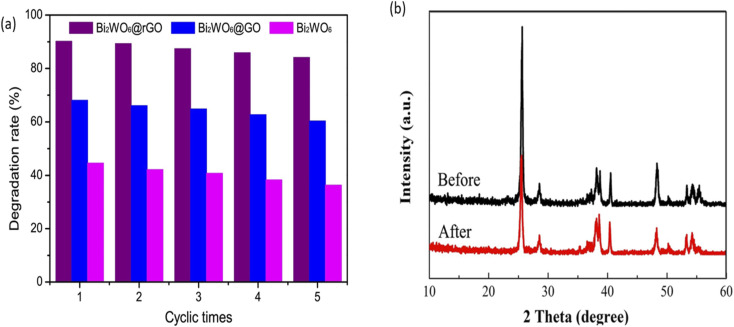
Reusability test for PEC degradation of rhodamine B using Bi_2_WO_6_@rGO (a) (reproduced from ref. 100 with permission from [Elsevier B.V.], copyright [2017]) and XRD spectra of Bi_2_WO_6_/TiO_2_ before and after PEC degradation of rhodamine B (b) (reproduced from ref. 101 with permission from [Elsevier B.V.], copyright [2018]).

An ideal photoanode for PEC degradation should possess good stability and reusability as this will project its potential for real-life applications. In order to assess the stability and reusability of photoanodes, structural and morphology characterisations of the photoanode before and after use can be done to check for any changes resulting from leaching of the material into solution. Additionally, comparison of percentage removal of the dye after several use of the same photoanode is a suitable method to establish the extent of reusability of the photoanode. A good number of proposed photoanodes for removal of rhodamine has demonstrated good stability and reusability. For example, in the PEC degradation of rhodamine B using a composite photoanode consisting of Bi_2_WO_6_ with reduced graphene oxide core shell (Bi_2_WO_6_@GO), the photoanode displayed good reusability after five runnings.^100^ As shown in Fig. 8, the change in the percentage removal after the fifth cycle was approximately 6% which confirmed that the electrode was stable. Likewise, TiO_2_/Bi_2_WO_6_ showed good stability in the PEC removal of rhodamine B which was affirmed from the XRD pattern of the photoanode after use. According to Fig. 8b, all the characteristic peaks of the material where still present after the use which suggested that electrode was stable.^101^ Though many reports on the stability of photoanodes in PEC degradation adopted the approach of assessing the change in the PEC efficiency after several use, it would be better if researchers also subject the solution to analysis such as Inductively Coupled Plasma Optical Emission Spectroscopy (ICP-OES) to further check the leaching of the electrode into solution.

## 5 Summary and future perspectives

Photoelectrocatalytic degradation has remained an efficient treatment option for the degradation of organics in wastewater. The process is environmentally benign and cost-effective. As summarized in Table 1, much effort has been channelled towards removing rhodamine B dye from an aqueous solution. The results obtained are quite fascinating and impressive. Obviously, different kinds of metal oxide semiconductors can be employed as photoanodes for the PEC system involving rhodamine B. However, the use of visible light-responsive photocatalysts is more enticing because they offer better PEC efficiency with reduced cost due to the use of simulated solar irradiation or direct sunlight.

**Table 1:** Summary of recent studies on PEC degradation of rhodamine B

Photoanode	Synthesis	PEC condition	Light source	Conc	% removal	Rate constant	Ref.
**Unmodified photoanodes**							
Bi_2_WO_6_/FTO	Spray pyrolysis	Potential: 0.7 V *vs.* SCE	500 W tungsten filament	30 mg L^−1^	94% after 4 h	1.08 × 10^−4^ s^−1^	84
		Pt wire and SCE as counter and reference electrode respectively					
Bi_2_S_3_/FTO	Radio frequency sputtering	Potential: 1.0 V *vs.* Ag/AgCl	100 W xenon lamp	10^−5^ M	89.2% after 80 min	0.0204 min^−1^	102
		Pt wire and Ag/AgCl as counter and reference electrode respectively					
MoS_2_/Ti	Hydrothermal	Potential: 0.5 V	300 W xenon lamp	1 mg L^−1^	100% after 2 h	0.00936 min^−1^	86
		Pt foil and Ag/AgCl as counter and reference electrode respectively					
BaHPO_4_/FTO	Electrodeposition	Current density: 13 mA cm^−2^	250 W xenon lamp	6.75 M	99% after 7 min	—	85
		Graphite rod and SCE as counter and reference electrode respectively					
SrHPO_4_/FTO	Electrodeposition	Current density: 10 mA cm^−2^	250 W xenon lamp	10 mg L^−1^	94.5% after 12 min	—	103
		Graphite rod and SCE as counter and reference electrode respectively					
MoS_2_/Ti	Hydrothermal	Potential: 0.3 V *vs.* Ag/AgCl	300 W xenon lamp	2 μM	100% after 4 h	0.0259 min^−1^	87
		Pt foil and Ag/AgCl as counter and reference electrode respectively					
Ru_*x*_Zn_1−*x*_O/Ti	Thermal decomposition	Potential: 2.5 V *vs.* SCE	—	20 mg L^−1^	97% after 2 h	—	88
		Ti wire and SCE as counter and reference electrode respectively					
ZnO/FTO	Electrodeposition	Pt wire and SCE as counter and reference electrode respectively	7 W UV lamp	10 mg L^−1^	99.98% after 30 min	0.2155 min^−1^	104
		Potential: 0.3 V					
Zn_3_(PO_4_)_2_/FTO	Electrodeposition	Current density: −1 mA cm^−2^	250 W xenon lamp	10 mg L^−1^	99% after 30 min	—	105
		Graphite rod cathode					

**Doped photoanodes**							
B–Co/TiO_2_	Anodisation	Potential: 0.6 V	254 nm W UV lamp	10 mg L^−1^	95.5% after 90 min	—	91
		Pt foil and Ag/AgCl as counter and reference electrode respectively					
Mn–N/TiO_2_/Ti	Thermal oxidation	Potential: 0.5 V	—	0.5 mg L^−1^	74.2% after 60 min	0.0229 min^−1^	94
		Pt foil and Ag/AgCl as counter and reference electrode respectively					
N–ZnO/FTO	Electrodeposition	Potential: 0.7 V	150 W lamp	1 μM	43% after 160 min	0.023 min^−1^	90
		Pt foil and Ag/AgCl as counter and reference electrode respectively					
N–TiO_2_/FTO	Chemical spray pyrolysis	Potential: 0.5 V	20 W UV lamp	0.5 mM	64% after 4 h	—	95
		Graphite cathode					
N–TiO_2_	Ion implantation	Pt mesh cathode	300 W tungsten-halogen lamp	80 mg L^−1^	43.2% after 80 min	0.011 min^−1^	106
		Potential: 2 V					
C–TiO_2_	Solvothermal	Stainless steel as cathode	—	10 mg L^−1^	37.8% after 70 min	—	107
ZnO/Ag	Chemical bath deposition	Ag rod as counter electrode	300 W Hg lamp	5 mg L^−1^	38% after 5 h	—	108
		Potential: 0.4 V					

**Heterostructured photoanodes**							
ZnFe_2_O_4_/TiO_2_/graphite	Sol–gel	Pt plate as cathode	500 W xenon lamp	20 mg L^−1^	99% after 30 min	0.278 min^−1^	109
		Potential: 15 V					
Ag_3_PO_4_/CNTs/Ni	Electrodeposition	Pt foil and SCE as counter and reference electrode respectively	500 W xenon lamp	5 mg L^−1^	95.44% after 12 min	—	110
		Potential: −0.1 V					
WO_3_/TiO_2_/FTO	Spray pyrolysis	—	Direct sunlight	1 mM	58.7% after 160 min	5.48 × 10^−7^ s^−1^	111
Bi_2_WO_6_@GO/ITO	Hydrothermal	Pt wire and SCE as counter and reference electrode respectively	500 W xenon lamp	5 mg L^−1^	90% after 4 h	—	100
		Potential: 1 V					
Bi_2_WO_6_/WO_3_/TiO_2_	Hydrothermal	Pt foil and Ag/AgCl as counter and reference electrode respectively	500 W xenon lamp	5 mg L^−1^	66.07% after 2 h	—	101
		Potential: 1 V					
Cu_2_O/TiO_2_	SILAR	Pt foil and Ag/AgCl as counter and reference electrode respectively	500 W xenon lamp	5 mg L^−1^	78% after 3 h	—	112
		Potential: 1 V					
BiVO_4_/TiO_2_	Hydrothermal	Pt wire and Ag/AgCl as counter and reference electrode respectively	300 W xenon lamp	10 mg L^−1^	93.9% after 5 h		113
		Potential: 6 V					
TiO_2_/Bi_2_MoO_6_	Solvothermal	Pt as counter electrode	500 W xenon lamp	20 mM	75% after 3 h	0.077 min^−1^	114
		Potential: 1 V					
Bi_2_O_3_/WO_3_	Hydrothermal	Pt foil and SCE as counter and reference electrode respectively	300 W xenon lamp	10 mg L^−1^	73.5% after 3 h	0.0073 min^−1^	115
		Potential: 1 V					
GO/Ag_3_PO_4_/Ni	Electrodeposition	Pt foil and SCE as counter and reference electrode respectively	—	8 mg L^−1^	97.01% after 12 min	—	116
		Potential: 0.3 V					
rGO/BiOI/rGO	Electrodeposition	Pt wire and SCE as counter and reference electrode respectively	300 W xenon lamp	5 mg L^−1^	80% after 5 h	0.322 h^−1^	117
		Potential: 1 V					
BiVO_4_/WO_3_	Dip coating	Pt wire and Ag/AgCl as counter and reference electrode respectively	500 W xenon lamp	5 mg L^−1^	94% after 3 h	—	118
		Potential: 2 V					
ZnO/CuWO_4_/FTO	Hydrothermal	Pt wire and Ag/AgCl as counter and reference electrode respectively	150 W mercury lamp	1 μM	82% after 3 h	0.0078 min^−1^	119
		Potential: 0.7 V					
Bi_2_MoO_6_/WO_3_	Hydrothermal and solvothermal	Pt foil and SCE as counter and reference electrode respectively	300 W xenon lamp	10 mg L^−1^	80.1% after 4 h	0.0069 min^−1^	120
		Potential: 1.5 V					
ZnO/ZnS/FTO	Hydrothermal	Pt wire and Ag/AgCl as counter and reference electrode respectively	100 W xenon lamp	0.1 mM	58% after 30 min	0.0249 min^−1^	121
		Potential: 0.5 V					

Nevertheless, most reported photoanodes for the removal of rhodamine B are modified either through doping or composite of two or more semiconductors with heterojunction. This is because photocatalysts are employed in their pristine form as photoanodes; they are more susceptible to spontaneous recombination of photogenerated electron–hole pairs, which tremendously reduces their PEC efficiency. Whereas, when modified appropriately, this problem can be circumvented through the promotion of efficient charge separation due to the formation of heterojunction or modification of band edge positions when doped, which results in higher PEC degradation efficiency of the dye. It is also very impressive to note that in some of the studies discussed, substantial removal of rhodamine B was achieved within a few minutes even with low applied potential, which confirmed the feasibility of adopting PEC oxidation for the treatment of textile effluents.

Nevertheless, there are some major issues that need to be addressed in future studies towards adopting PEC treatment for real-life applications. Firstly, in many of the reported work, the researchers did not report actual mineralisation of the rhodamine B molecule through total organic carbon (TOC) or determination of intermediate products. It is important to note that some of these intermediates could be more toxic than the parent rhodamine B molecules which necessitate conducting toxicity tests using bacteria strains. In addition to the toxicity of the intermediate products that can be challenging, formation of intermediate products could also increase the energy consumption of the PEC process. Computation of energy consumption within the process using the TOC values could provide appropriate insights on this.

Secondly, it is also troubling to note that the full experimental setup was not probably described in these studies, which makes it difficult for fair comparisons with other literature. In future studies, full description of the experimental setup is recommended. This should include the type of reactor use, the intensity of the light source, pollutant concentration, volume of working solution, the applied potential, distance of the photoanode from light source and other essential parameters that will facilitate easy reproducibility of the results and fair comparison with existing literature.

Additionally, the majority of the studies were conducted using simulated rhodamine B contaminated water but not real industrial effluent. Hence, it is recommended that future researchers pay more attention to the determination of the rate of mineralisation of rhodamine B and not just discolouration. Also, particular efforts should be directed to using real effluents for degradation studies. Overall, the PEC oxidation process has shown remarkable potential towards the treatment of wastewater laden with organic dyes such as rhodamine B.

## Conflicts of interest

There are no conflicts to declare.

## Supplementary Material
